# Enhanced peripheral nerve regeneration by the combination of a polycaprolactone tubular prosthesis and a scaffold of collagen with supramolecular organization

**DOI:** 10.1002/brb3.145

**Published:** 2013-05-30

**Authors:** Luiz G Maturana, Amauri Pierucci, Gustavo F Simões, Mateus Vidigal, Eliana A R Duek, Benedicto C Vidal, Alexandre L R Oliveira

**Affiliations:** 1 Department of Structural and Functional Biology, University of Campinas – UNICAMPCampinas, Brazil; 2 Department of Morphology, Federal University of Mucuri and Jequitinhonha Valley – UFVJMDiamantina, Brazil; 3 Department of Materials Engineering, UnicampCampinas, Brazil

**Keywords:** Biomaterials, collagen, nerve regeneration, polarization microscopy, tubulization

## Abstract

The purpose of this study was to investigate the influence of implanting collagen with a supramolecular organization on peripheral nerve regeneration, using the sciatic nerve tubulization technique. For this purpose, adult female Sprague Dawley rats were divided into five groups: (1) TP – sciatic nerve repaired with empty polyethylene tubular prothesis (*n* = 10), (2) TPCL – nerve repair with empty polycaprolactone (PCL) tubing (*n* = 8), (3) TPCLF – repair with PCL tubing filled with an implant of collagen with a supramolecular organization (*n* = 10), (4) AG – animals that received a peripheral nerve autograft (*n* = 8), and (5) Normal nerves (*n* = 8). The results were assessed by quantification of the regenerated fibers, nerve morphometry, and transmission electron microscopy, 60 days after surgery. Immunohistochemistry and polarization microscopy were also used to analyze the regenerated nerve structure and cellular elements. The results showed that the AG group presented a larger number of regenerated axons. However, the TPCL and TPCLF groups presented more compact regenerated fibers with a morphometric profile closer to normal, both at the tube midpoint and 2 mm distal to the prosthesis. These findings were reinforced by polarization microscopy, which indicated a better collagen/axons suprastructural organization in the TPCLF derived samples. In addition, the immunohistochemical results obtained using the antibody anti-p75NTR as a Schwann cell reactivity marker demonstrated that the Schwann cells were more reactive during the regenerative process in the TPCLF group as compared to the TPCL group and the normal sciatic nerve. Altogether, the results of this study indicated that the implant of collagen with a supramolecular organization positively influenced and stimulated the regeneration process through the nerve gap, resulting in the formation of a better morphologically arranged tissue.

## Introduction

The tubulization technique has been used for several years as an experimental model to study peripheral nerve regeneration (Fields et al. 1989; Lundborg et al. 1994, 2004; Oliveira et al. 2004). It has also been used as an alternative to autografting in certain situations, such as for small gaps between sectioned stumps or the need to repair digital nerves (Lohmeyer et al. 2009). Thus, the development of bioreabsorbable polymers has opened an entire new field of investigation, allowing for the perspective of using the tubulization technique to repair larger nerves such as the median, the ulnar, or even brachial plexus branches.

Associated with the use of new biocompatible polymers, the possibility of bridging stumps with extracellular matrix molecules inside the tubular prosthesis has made it possible to significantly improve axonal regeneration. This fact is associated with an increased success of restoring movement in a shorter period of time. More recently, it has been proposed that aligned scaffolds made of natural or synthetic materials, could improve axonal growth and facilitate the correct reattachment between the stumps inside the tube (Verdú et al. 2002; Kijeńska et al. 2012; Wang et al. 2012). Such scaffolds would also allow an increase in the gap between the proximal and distal stumps, which is one of the main advantages of the autograft repair approach.

The use of functional molecules that may have autoassembling characteristics can facilitate the construction of organized scaffolds. Thus, the architecture of the collagen fibers plays a critical role in determining the biomechanical behavior of the extracellular matrix, and the alignment and organization of its fibers depend on the function of the tissue in which they are found. Therefore, the collagen fibers of the extracellular matrix derived from tendons and ligaments are highly aligned to the long axis of the whole structure (Badylak et al. 2009). Nevertheless, the fibers and/or bundles of collagen tendons are not arranged in a flat structure, and the pattern of waves reflects the helical organization of the collagen fibers/bundles. Thus, the orientation of the tendon fibers can be considered as a complex structure with supramolecular organization (Vidal 2003; Vidal and Mello 2010).

Axial sections of bovine tendons treated with acetic acid and examined under polarized light support the supra-organization of helical bundles of collagen in these tendons, and a similar organization has been described in rat tendons (Vidal 2003).

The organization and state of molecular aggregation of a biological implant are important factors that provide a suitable environment for axonal guidance and regeneration, and a naturally oriented protein can facilitate axonal growth and be degraded more efficiently (Fields et al. 1989; Labrador et al. 1998; Ceballos et al. 1999). In contrast, collagen, when subjected to different treatments, does not reproduce the helically organized pattern of fibers (Oliveira et al. 2005).

The present authors previously showed that Schwann cells cultured on a naturally aligned collagen substrate expressed higher levels of the low-affinity receptor for neurotrophins (p75NTR) and for S100 (Pierucci et al. 2009). Also, cell orientation was enhanced when the cultures were established in the organized collagen substrate. It is possible that such a scaffold may provide a better support for regenerating axons if inserted into the gap between the nerve stumps using the tubulization technique. Thus, polystyrene scaffolds, produced so as to replicate the basal lamina of peripheral nerves, lead to a neurite alignment parallel to the structure of the basal-lamina like tubes obtained in the preparation (Karlsson et al. 2011).

Another interesting approach to obtain longitudinal organization of the artificial implant was proposed by Lu et al. (2005), who used capillary channel fibers containing grooves, made from poly(l-lactic acid) (PLA) and polyethylene terephthalate (PET). Micro-structured biomaterial filaments were also used in vitro and in vivo to induce the formation of Büngner bands and increase the gap between the stumps after peripheral nerve repair using tubulization. For this purpose, resorbable polycaprolactone (PCL) filaments were formed by a melting extrusion technique using capillary size molds and coated with poly-d-lysine and laminin (Ribeiro-Resende et al. 2009).

Based on the information reported above, the present authors developed a strategy to increase the axonal regeneration process after using the tubulization technique, by combining the use of a tubular prosthesis made of PCL with a supraorganized collagen foam implant placed inside the tube, and the results were compared with the use of the autograft technique. Morphological and morphometrical analyses lead to the conclusion that the above mentioned approach increased the Schwann cell reactivity, as seen by the S100 and p75NTR expressions. Also the myelin thickness and the “g” ratio data demonstrated a better reorganization of the regenerated nerves when subjected to the proposed experimental approach.

## Materials and Methods

### Preparation of collagen with supra-molecular organization

The collagen samples were extracted from the calcaneal tendon of cattle using a patented technique (#P.I.97015709, B. C. Vidal). Fragments of bovine tendon samples were cleaned and immersed in a solution containing 5% acetic acid, 0.01% HCl, and 1 mg of pepsin/g of tendon, maintaining at 7–10°C for 24 hours. The dissolved collagen was filtered and the fibers reconstituted by adding a 5% NaCl solution. The fibers thus obtained were dialyzed against distilled water at 5°C in a 6-mm-diameter tube, exchanging the water every 24 hours. One and a half liters of water were needed to acquire a total of 200 g of collagen with a supra-molecular organization (Oliveira et al. 2005). This was accomplished examining the collagen gel after dialysis with polarized light microscopy (PLM) to detect the reconstituted fibers birefringence. Furthermore, gel was extruded and the helical arrangement of collagen fibrils was detected by their birefringence by PLM (see Vidal 1995).

### Construction of the prosthesis for tubulization

The tubular prosthesis was made using the solvent technique, as previously described (Pierucci et al. 2008). The polymer solution was prepared by adding 1.65 g of PCL (molecular weight = 100 kDa; PURAC Biochem, Gorinchem, the Netherlands) to 33 mL of solvent (dichloroethane; Merck, Darmstadt, Germany). After initial solubilization, the solution was left at room temperature for 12 hours to complete the homogenization. The next day, the solution was placed on a plate contained in a glass vat of which the middle part was saturated with solvent. The system was left for 12 hours to allow for complete evaporation of the material, and the membrane then removed from the glass vat and placed in a vacuum chamber for 5 minutes, so that eventual residues of the solvent did not remain on the membranes. The membranes were then rolled onto 1.6-mm-diameter pins (Evans et al. 1999) and cut into 10 mm lengths.

### Animals and experimental groups

Adult female Sprague Dawley rats (NTac Unib:SD) were used in the present study, divided into the following groups:

1. Group 1: TP – animals treated with an empty polyethylene tubular prosthesis (*n* = 7 for morphometry; *n* = 3 for immunohistochemistry and polarization microscopy).
2. Group 2: TPCL – animals treated with an empty PCL tubular prosthesis (*n* = 5 for morphometry; *n* = 3 for immunohistochemistry and polarization microscopy).
3. Group 3: TPCLF – animals treated with a PCL tubular prosthesis filled with a collagen implant with supra-molecular organization (*n* = 7 for morphometry; *n* = 3 for immunohistochemistry and polarization microscopy).
4. Group 4: AG – animals that received a peripheral nerve autograft (*n* = 5 for morphometry; *n* = 3 for immunohistochemistry and polarization microscopy).
5. Normal nerves (*n* = 5 for morphometry; *n* = 3 for immunohistochemistry and polarization microscopy).

### Surgical procedure for tubulization

Following anesthesia with Kensol (xylasine, Köning, Argentina, 10 mg/kg) and Vetaset (Cetamine, Fort Dodge, IA, 50 mg/kg, i.p.), the animals underwent trichotomy of the left thigh. The skin was incised, and the sciatic nerve exposed by retracting the musculature and then transected. After retracting the stumps, the nerve was repaired according to the experimental groups. For the TP group, the proximal and distal stumps of the sciatic nerve were introduced into an empty polyethylene tubular prosthesis, and fixed to the ends of the prosthesis with a surgical suture through the epineurium of the nerve, maintaining the stump alignment and leaving a gap of 6 mm between them. The same procedures were followed for the TPCL group, except that the tubular prosthesis was made of PCL. The TPCLF group also followed the same tubulization procedures described above, except that before fixation of the distal stump to the end of the PCL tube, a collagen implant with supra-molecular organization was inserted into the middle part of the tube. For the AG group, autografting was carried out right after the separation of the proximal and distal stumps. An approximately 6–7 mm segment of the sectioned nerve was reversed and reattached to the proximal and distal stumps using two surgical stitches passing through the epineurium (neurorrhaphy), maintaining the continuity of the sciatic nerve. Following the nerve repair procedures, the muscular plane was sutured with 7-0 silk and the skin closed with 3 surgical stitches (mononylon 4-0, Ethicon, São José dos Campos, Brasil). The animals were kept in a vivarium for a 60-day period, receiving food and water ad libitum.

### Sacrificing of the animals and processing of the specimens for transmission electron microscopy

After the 60-day survival period, the animals from all the groups were anesthetized with Kensol (xylasine, 10 mg/kg) and Vetaset (Cetamine, 50 mg/kg, i.p.), and submitted to thoracotomy followed by transcardiac perfusion with the aid of a peristaltic infusion pump. Initially, in order to wash the vessels and organs, the animals were perfused with 150 mL of a buffered saline solution (0.9% NaCl in 0.1 mol/L phosphate buffer [PB], pH 7.4). They were then fixed by infusing 300 mL of a solution containing glutaraldehyde (2%) and paraformaldehyde (1%) in 0.1 mol/L PB, pH 7.4. After fixation, the set containing the regenerated nerve inside the tube, a nerve fragment 2 mm distal to the tube and the autograft were dissected out and immersed in the same fixative solution for 12 hours at 4°C. After this period, these elements were washed in 0.1 mol/L PB, pH 7.4 and dissected under a microscope such that the proximal and distal stumps were separated. The fragments were individually placed into vials containing 0.1 mol/L PB, pH 7.4, which were postfixed for a period of 2 hours in a 1% solution of osmium tetroxide diluted in 0.1 mol/L PB, pH 7.4. Following postfixation, the fragments were washed in distilled water and dehydrated in an increasing series of acetone and then embedded in resin (Durcupan ACS, Fluka, Germany), positioned for transverse sectioning. The blocks were trimmed and semi-thin sections (0.5 μm), from the regenerated nerves at the tube midpoint, were obtained and stained with 0.25% toluidine blue for light microscopy observation. In sequence, representative regions were selected and the blocks retrimmed in order to produce ultrathin sections (500Å; Ultracut, Leica, Wien, Germany) which were collected on copper grids (200 mesh, EMS, Philadelphia, PA). After contrasting using uranyl acetate (EMS) and lead citrate (EMS), the specimens were observed under a Zeiss Leo 906 (Carl Zeiss, Oberkochen, Germany) transmission electron microscope operating at 60 kV.

### Morphometry and count of the regenerated fibers

For the morphometric analysis that was carried out at the tube or autograft midpoint, the following parameters were considered: number of regenerated myelinated axons, thickness of the myelin sheath (MT), and the “g” ratio (GR). The study of the response of the Schwann cells to the nerve repair was based on the values for MT and GR. For this purpose, four fields were sampled in each regenerated nerve and used to measure the diameters of the fibers and axons. The MT was calculated from the difference between the diameter of the fibers and their respective axons divided by 2 (Mayhew and Sharma 1984). The GR consists of a numeric value that provides information about the myelination state in relation to the size of the axon (axon diameter/fiber diameter), which is considered normal when close to 0.7. The morphometric analysis was carried out using a computerized system running the software Image Tool 3.00 (UTHSCSA, San Antonio, TX).

To count the myelinated axons, four representative fields from each regenerated nerve were digitized with the aid of a digital video camera (Olympus U-Cmad-2 Philadelphia, PA) connected to an Olympus BX60 microscope (×1000). The total number of fibers was estimated taking into account the total area of the respective regenerated nerve.

In order to confirm that the regenerated axons reached the distal stump, a histological study of the distal stump, 2 mm distal to the tube end, was carried out.

### Animals and experimental groups for immunohistochemistry and polarizing microscopy

For immunohistochemistry and polarization microscopy, additional animals were operated (*n* = 3 for each group) for composing the same groups previously mentioned.

### Sacrificing of the animals and processing of the specimens for immunohistochemistry

After the predetermined survival time, the animals were perfused according to the procedure used for transmission electron microscopy, but after perfusion with 300 mL of saline, a subsequent perfusion with a 10% formalin solution in 0.1 mol/L PB, pH 7.4 was carried out.

After fixation, the set containing the regenerated nerve inside the tube was dissected and immersed in the same fixative solution for 12 hours, maintained at a temperature of 4°C. After this period, the elements of the samples were washed in 0.1 mol/L PB, pH 7.4, and dissected under the microscope. The nerves were placed individually into vials containing a 20% sucrose solution in 0.1 mol/L PB, pH 7.4 and maintained for 12 hours, before immersing in tissue-tek (Milles Inc., Torrance, CA) and freezing in *n*-hexane (Merck). The frozen samples were maintained in liquid nitrogen at −40°C. Frozen longitudinal 12-μm-thick sections were obtained in a cryostat (Microm, Walldorf, Germany), transferred to gelatinized slides and stored at −20°C until used. For the immunohistochemical analysis, the specimens were taken out of the freezer and allowed to reach room temperature. They were then immersed in 0.1 mol/L PB, pH 7.4, and incubated in a solution containing 1% bovine albumin (BSA) in 0.1 mol/L PB, pH 7.4, for 1 hour. After three washes in 0.1 mol/L PB, pH 7.4, the primary antibodies were applied: (1) rabbit anti-S-100 – marker for the calcium carrier protein localized throughout the cytoplasm of Schwann cells (DAKO, Glostrup, Denmark); (2) rabbit anti-p75NTR – low-affinity receptor for the nerve growth factor (NGF) and other neurotrophins (brain-derived neurotrophic factor, NT3/4) (Santa Cruz, Dallas, TX); (3) rabbit anticollagen type IV (Santa Cruz), rabbit antilaminin (expressed by Schwann cells, being located in their basement membrane) (Santa Cruz), and mouse antineurofilament (axons cytoskeleton protein) (DAKO). All the antibodies were incubated for 2 hours at 4°C. In sequence, after washing with 0.1 mol/L PB, pH 7.4, the respective secondary antibodies conjugated with Cyanine (CY)-2 or CY-3 were applied for 45 minutes at room temperature. The slides were washed in 0.1 mol/L PB, pH 7.4, and mounted in glycerol/PB (3:1), being observed under a fluorescence microscope (TS100; Nikon, Otawara, Japan). For quantification of the images, five representative areas of each specimen were measured with respect to the integrated density of pixels using the ImageJ software (version 1.33u, National Institute of Health, Bethesda, MD). A mean value per animal was used to calculate the whole-group mean, and the averages of each group were analyzed and statistically compared.

### Polarizing microscopy

The same groups, amounts of sample, procedures for fixing the specimens, and obtaining the frozen sections referred to above in the previous technique were used for polarization microscopy.

After reaching room temperature, the slides with longitudinal sections of regenerated nerves were mounted in distilled water and observed under the polarizing microscope (Olympus BX51-P BX2). Nerves are negative birefringence (ne > no) with respect to their long axis due to the acyl group of lipid staking perpendicular to the nerve axis, this mimic a smectic liquid crystal (Vidal et al. 1980; Vidal 2010). So, to detect nervous fiber and their orientation in sections it was used the rotating stage of the microscope to visualize the birefringence brilliance dependent on the relative angle of the nervous fibers to the crossed analyzer and polarizer. To study the myelin sheath the compensators according Senarmont 1/4λ and/or Braceköler 1/10λ were employed (Vidal et al. 1980). This microscope allows one to change the inclination angle of the slide, and hence that of the tissue in relation to the analyzer and the polarizer. Depending on the angle of the slide, the desired birefringence compensation could be obtained, increasing or decreasing the brightness intensity of the collagen (−45°) or of the myelin sheath (+45°). The images captured were analyzed using the image analyzer Image-Pro Plus 6.3, Media Cybernetics, Inc. (Silver Spring, MD).

### Statistical analysis

The data are presented as the mean ± SEM and were analyzed using the one-way ANOVA followed by Bonferroni post hoc test, for multiple comparisons at *P* < 0.05 (*), *P* < 0.01 (**), and *P* < 0.001 (***).

## Results

### Characterization of the collagen with supramolecular organization

**Figure 1:**
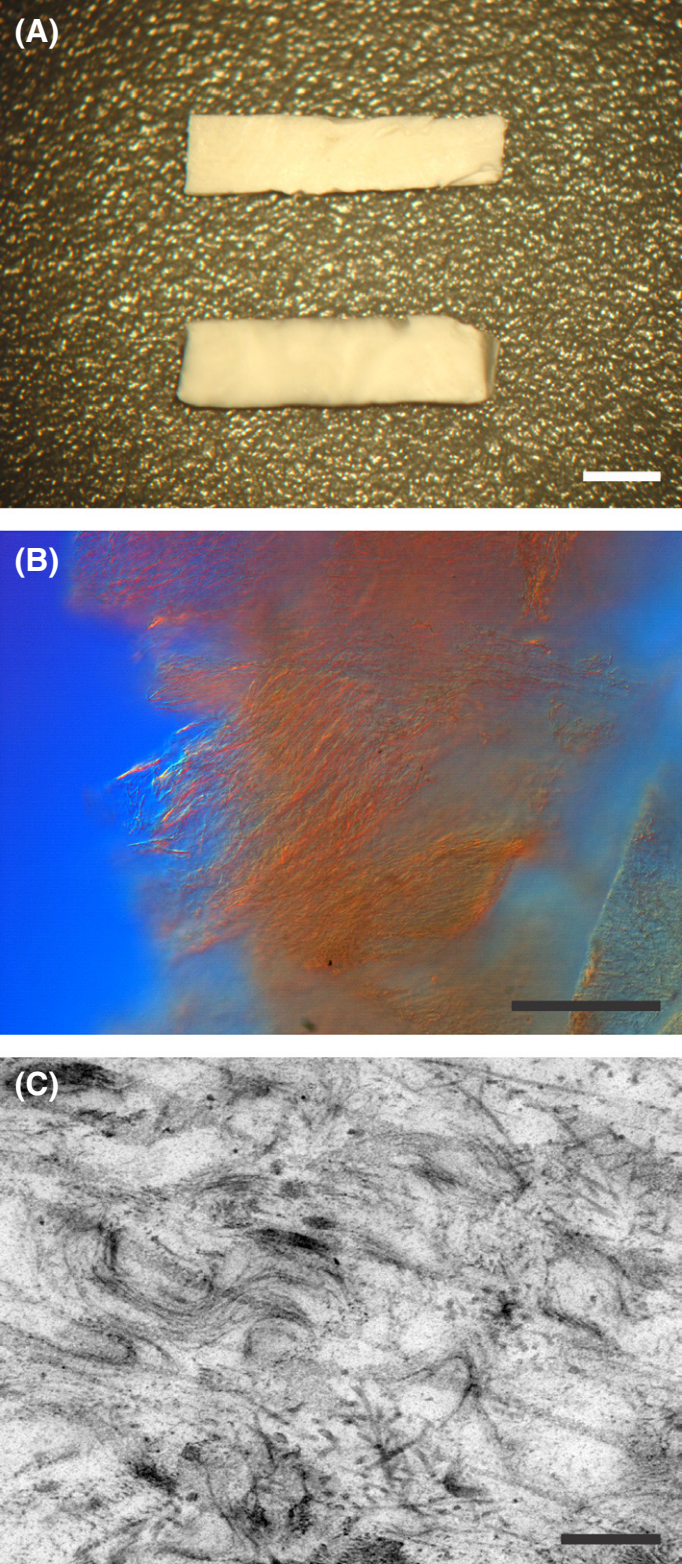
Characterization of the collagen with supra-molecular organization. (A) Fragment of collagen used for a preimplant in vivo. Scale = 1 mm. (B) Sample of collagen with supra-molecular organization observed under the polarization microscope. Scale = 100 μm. (C) Transmission electron micrograph of the collagen implant showing the parallel organization of the fibers. Scale = 1 μm.

Macroscopically, the implants were shaped in a prismatic form and showed a white coloration with a spongy aspect and soft texture (Fig. 1A). The material proved to be hydrophilic in contact with saline, assuming the aspect of a gel without, however, dissociation. When observed under the polarization microscope, the collagen fibers were proven to be arranged parallel to each other with a high degree of alignment at the different depth levels (Fig. 1B). This aspect was also seen under transmission electron microscopy (Fig. 1C). A 3D electron tomography reconstruction video of the collagen implant is presented as Movie S1.

### Microscopic aspect of the regenerated nerves

**Figure 2:**
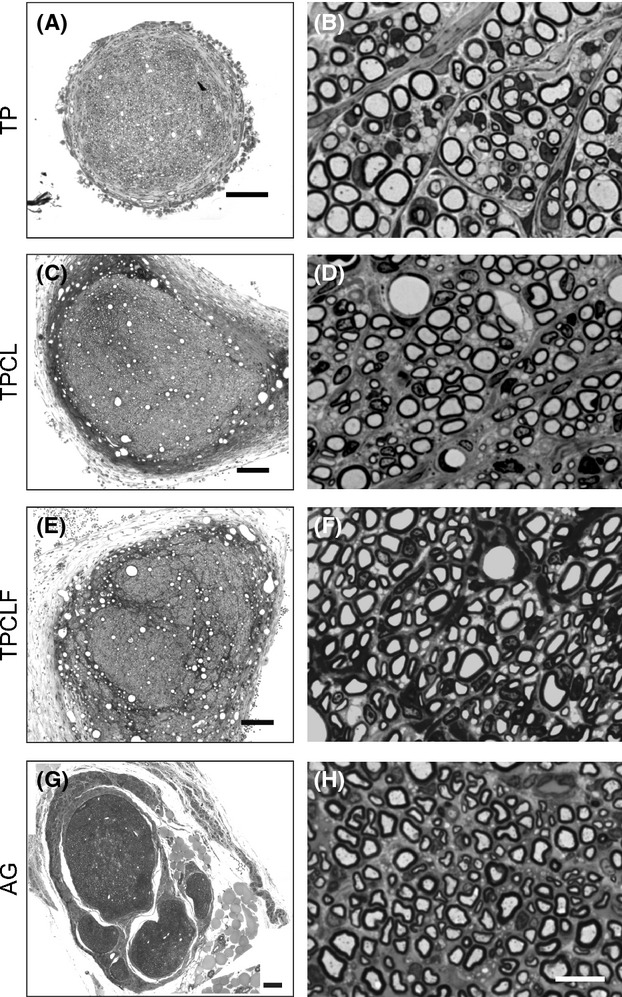
Panoramic view of regenerated nerves from the different experimental groups observed under light microscopy 60 days after tubulization. TP, implant of an empty polyethylene tube (A and B), TPCL, implant of an empty polycaprolactone tube (PCL) (C and D), TPCLF, implant of a PCL tube filled with a collagen implant with supra-molecular organization (E and F), and AG, peripheral nerve autograft (G and H). Scale = 100 μm.

In cross sections of the regenerated nerves from the TP, TPCL, and TPCLF groups, the presence of an epineurium was evident and presented different thicknesses and number of blood vessels. Also, when the nerve repair was performed with PCL, a greater number of blood vessels were evident, as seen in Figure 2C and E. The samples had a normal cylindrical shape and the formation of fascicules containing nerve fibers was more evident in the tubulization-derived samples.

In the TPCLF group, no trace of the collagen implant with supramolecular organization could be detected, indicating the complete absorption/remodeling of such material in vivo. In the AG group, the epineurium showed a more complex organization, even containing adipose deposits, and presented large groups of fibers arranged outside the main structure of the nerve, indicating the sprouting of fibers (Fig. 2G).

**Figure 3:**
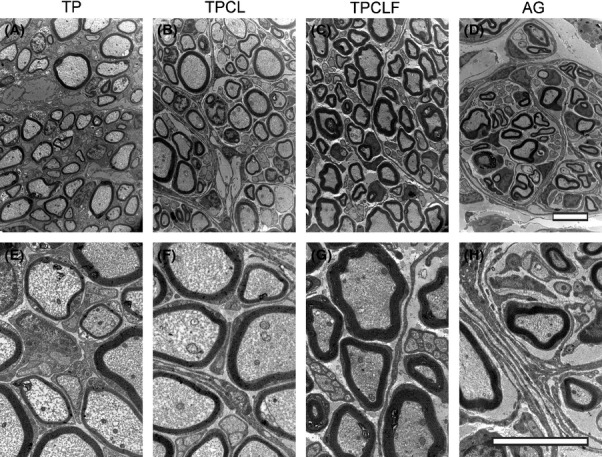
Transmission electron microscopy of the different experimental groups 60 days after tubulization. TP, implant of an empty polyethylene tube (A and E), TPCL, implant of an empty polycaprolactone tube (PCL) (B and F), TPCLF, implant of a PCL tube filled with a collagen implant with supra-molecular organization (C and G), and AG, peripheral nerve autograft (D and H). Scale = 5 μm.

In the TP group, a microenvironment composed of axons showing a slender myelin sheath was observed by transmission electron microscopy. The TPCL group revealed a better organized endoneural microenvironment, containing numerous mini-fascicles intermingled with collagen type I fibers. The thickness of the myelin sheath was shown to be greater in these axons in comparison to the previous group. In the TPCLF group, the compactness of the nerve fibers in the mini-fascicles was more evident, indicating a better organization of the microenvironment in addition to having a reduced amount of extracellular matrix. The thickness of the myelin sheath of this group proved to be the largest as compared to all the other groups. The microenvironment of the AE group expressed a large collection of extracellular matrix, containing, in some areas, more elements from the extracellular matrix than nerve fibers and the axons were organized into large fascicles. The myelinated fibers displayed variable diameters as well as variable thicknesses of the myelin sheath (Fig. 3).

**Figure 4:**
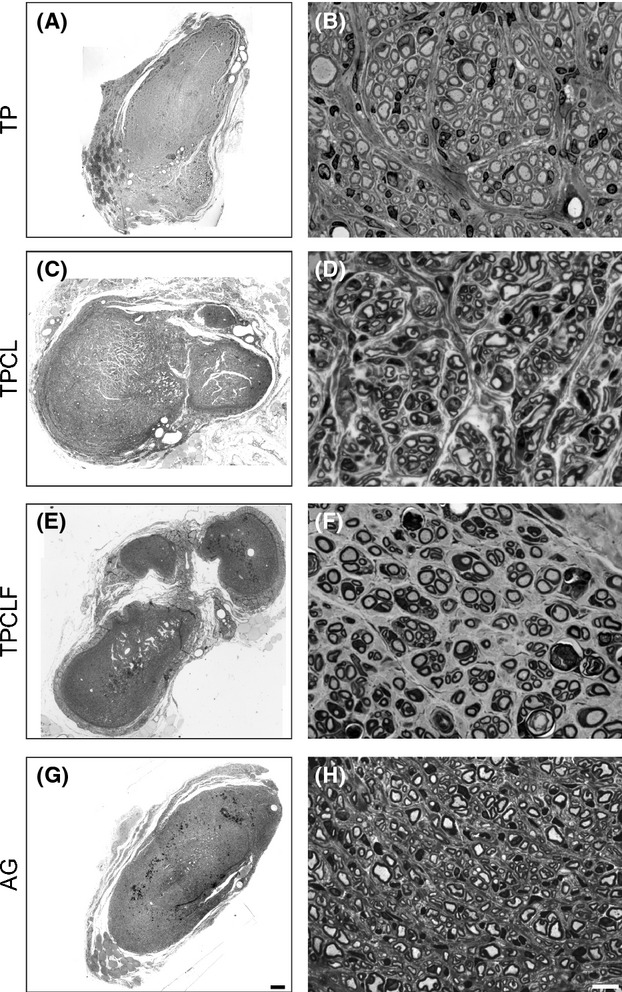
Panoramic view of regenerated nerves 2 mm distal to the tube end in the different experimental groups, observed under light microscopy 60 days after tubulization. TP, implant of an empty polyethylene tube (A and B), TPCL, implant of an empty polycaprolactone tube (PCL) (C and D), TPCLF, implant of a PCL tube filled with a collagen implant with supra-molecular organization (E and F) and, AG, peripheral nerve autograft (G and H). Scale = 100 μm (A, C, E, G) and 10 μm (B, D, F, H).

In order to ensure that the axons observed at the tube midpoint reached the distal stump, samples were collected 2 mm distal to the tube end. The results showed similar axonal regeneration as observed at the tube midpoint (Fig. 4).

### Quantification and morphometric analysis of the regenerated nerve fibers under light microscopy at the tube midpoint

**Figure 5:**
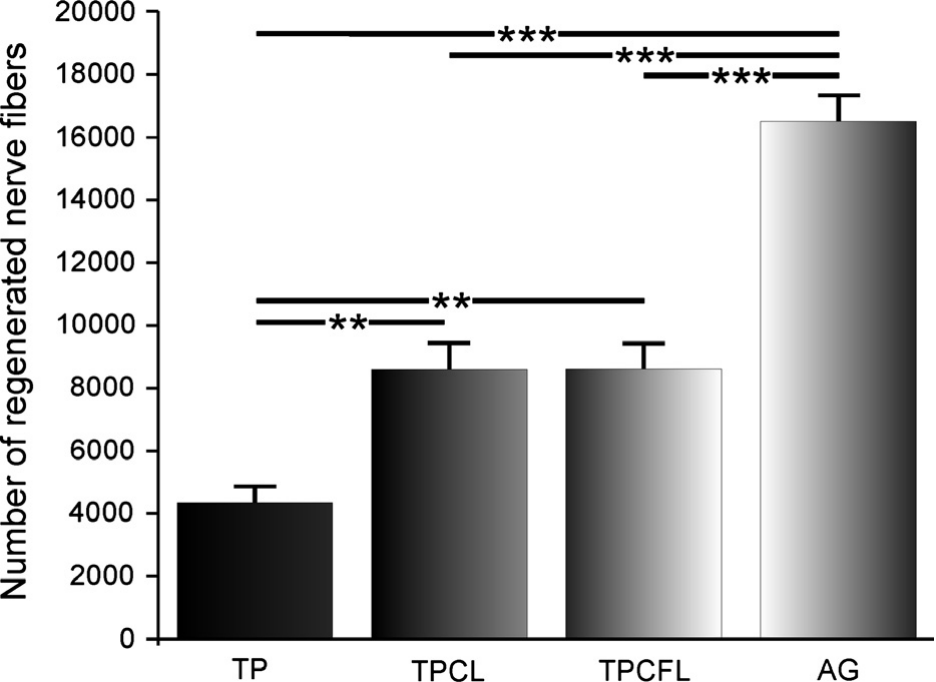
Estimation of the number of regenerated fibers in the different experimental groups 60 days after surgery (**P < 0.01 and ***P < 0.001). TP, implant of an empty polyethylene tube, TPCL, implant of an empty polycaprolactone tube (PCL), TPCLF, implant of a PCL tube filled with a collagen implant with supra-molecular organization, and AG, peripheral nerve autograft.

The estimate for the total number of regenerated nerve fibers for the AG group was significantly greater than for the other experimental groups (*P* < 0.001). Overall, the TP group showed the smallest number of regenerated fibers in relation to the other groups (AG – 16,454.45 ± 820.84; TP – 5,257.91 ± 506.43; TPCL – 9,291.29 ± 847.41; and TPCLF – 9,605.04 ± 813.18, Normal – 7,414.01 ± 136.96, mean ± SEM, *P* < 0.01). There were no differences between the number of regenerated axons in the TPCL and TPCLF groups (Fig. 5).

**Figure 6:**
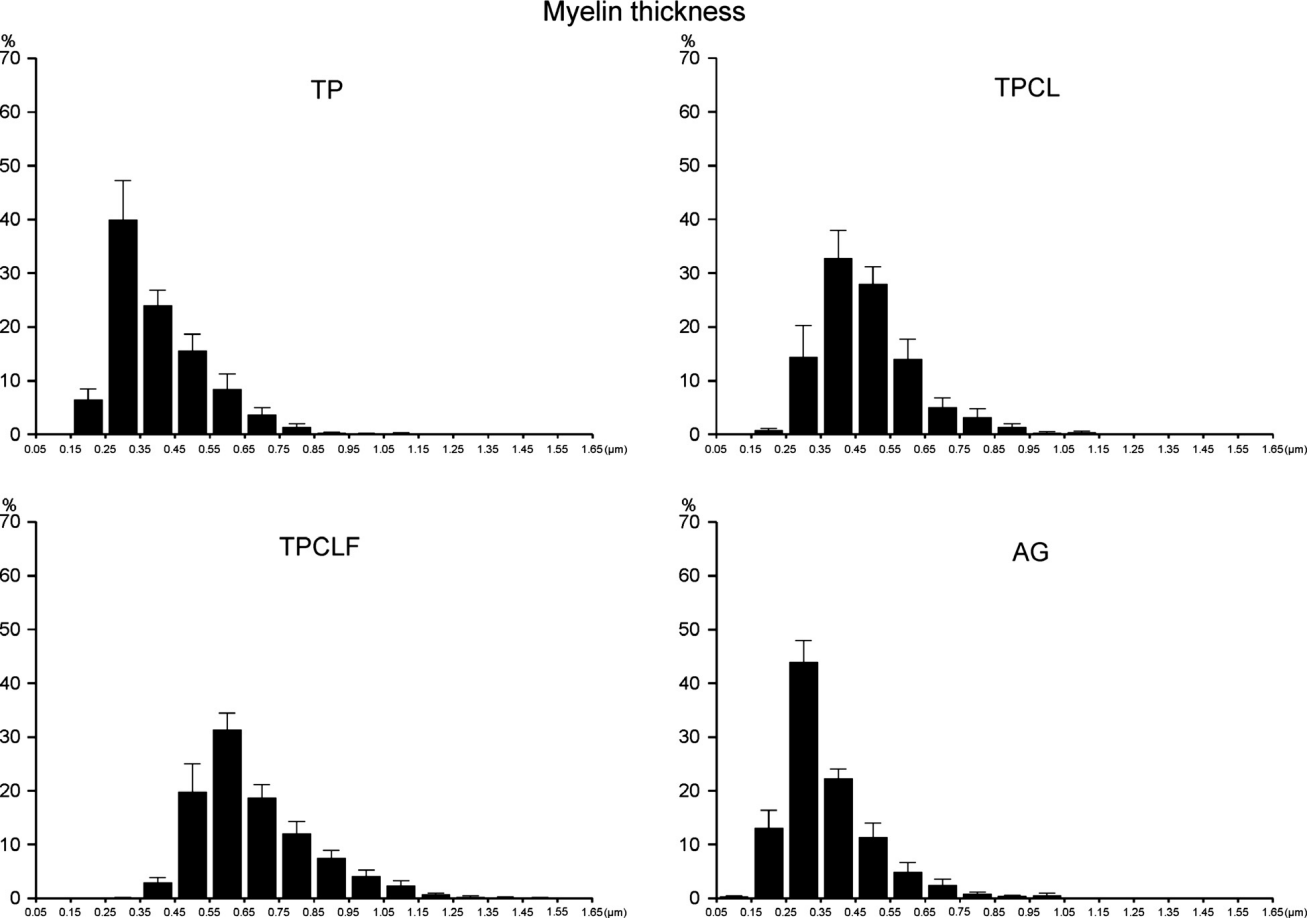
Frequency distribution of the thickness of the myelin sheath (MT) of the regenerated nerves in the different experimental groups 60 days after surgery. TP, implant of an empty polyethylene tube, TPCL, implant of an empty polycaprolactone tube (PCL), TPCLF, implant of a PCL tube filled with a collagen implant with supra-molecular organization, and AG, peripheral nerve autograft.

Regarding the MT measurements, the frequency distribution analysis showed that the AG and TP groups had statistically significantly more fibers with a reduced myelin thickness than those of the TPCL and TPCLF groups. Also, the TPCLF group presented a thicker myelin sheath than all the other groups, except in the frequency interval from 0.46 to 0.55 μm, where there was no significant difference (AG – 0.31 ± 0.05; TP – 0.35 ± 0.04; TPCL – 0.43 ± 0.05; and TPCLF – 0.62 ± 0.05, Normal – 1.12 ± 0.1, mean ± SEM, *P* < 0.01). These findings indicated a more active myelinating behavior of the Schwann cells in the TPCLF group (Fig. 6).

**Figure 7:**
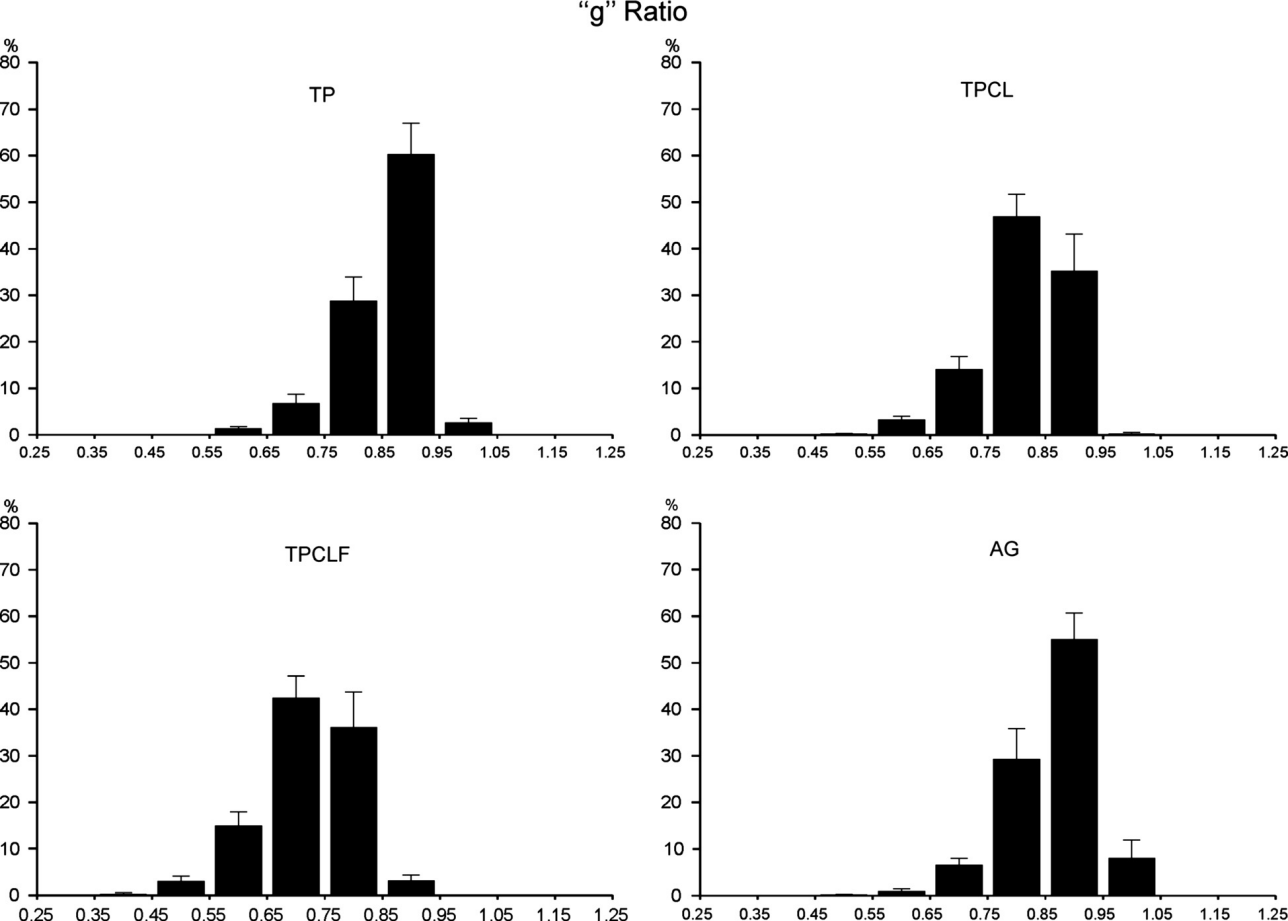
Frequency distribution of the “g” ratio of the experiments carried out with the different experimental groups, 60 days after surgery. TP, implant of an empty polyethylene tube, TPCL, implant of an empty polycaprolactone tube (PCL), TPCLF, implant of a PCL tube filled with a collagen implant with supra-molecular organization, and AG, peripheral nerve autograft.

In a similar way, the “g” ratio was closer to the normal values in the TPCLF group, as the result of a more balanced relationship between the diameter of the myelinated axons and the diameter of the axons themselves. Contrarily, the data from the TP, TPCL, and AG groups indicated a shift to an increased presence of thinner myelinated axons, consistent with hypomyelination (AG – 0.81 ± 0.02; TP – 0.80 ± 0.02; TPCL – 0.76 ± 0.07; and TPCLF – 0.67 ± 0.02, Normal – 0.71 ± 0.01, mean ± SEM, *P* < 0.01; Fig. 7).

### Immunohistochemistry

**Figure 8:**
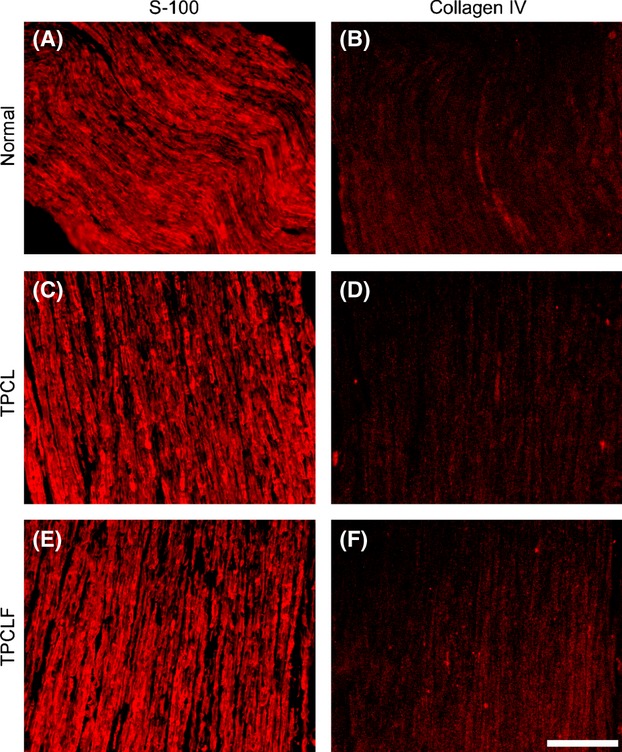
Immunohistochemistry using the antibodies anti-S-100 (A, C, and E) and Collagen IV (B, D, and F) of the normal group (A and B), TPCL (C and D) and TPCLF (E and F), 60 days postsurgery. Scale = 100 μm.

Longitudinal sections of the regenerated nerves were immunostained with the antibody anti-S-100, and an equally intense labeling was observed for all groups. For the TPCL and TPCLF groups the nerve fibers displayed the standard parallel disposition to the long axis of the nerve, consistent with normal nerves with a parallel pattern of wavy fibers (Fig. 8).

**Figure 9:**
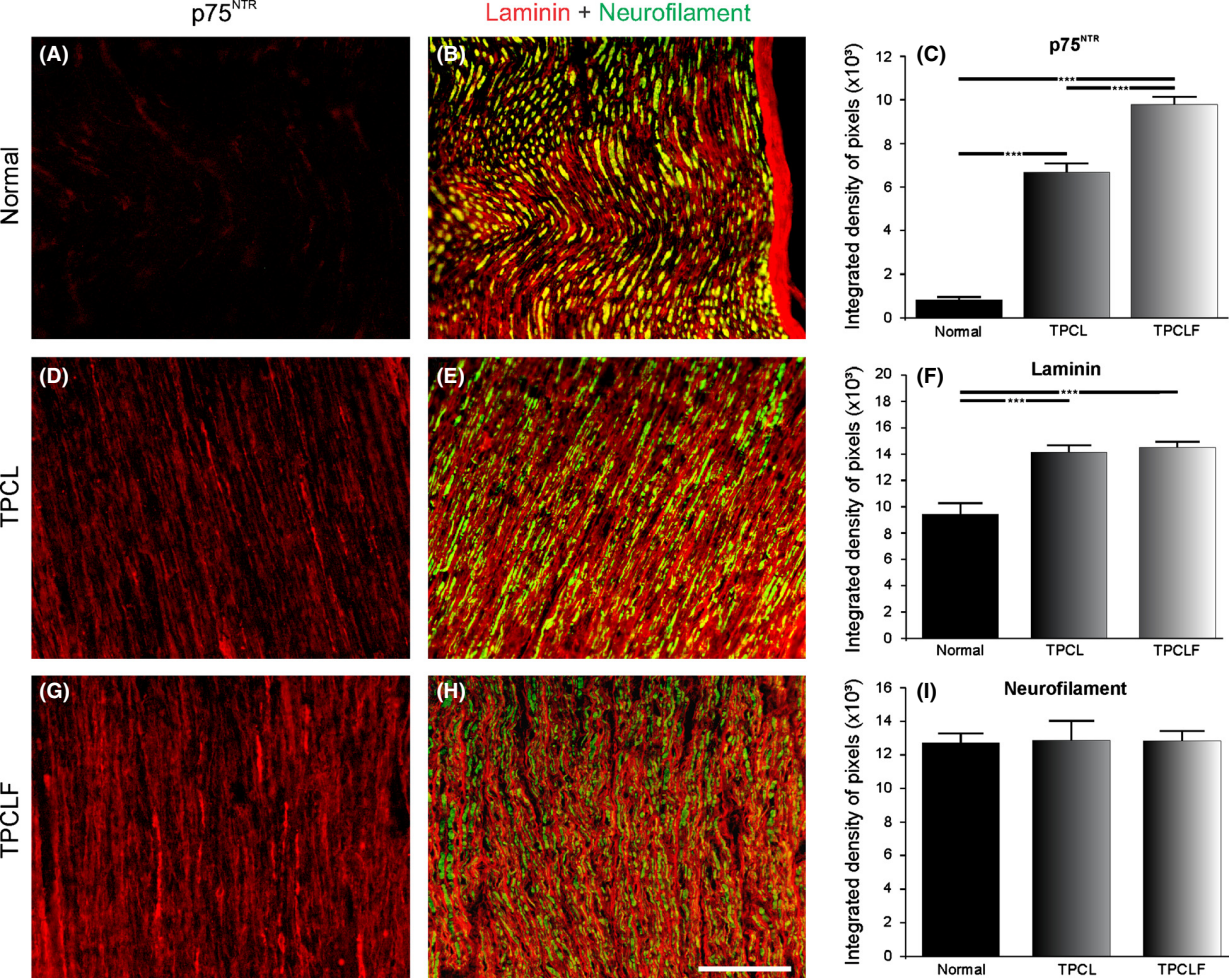
Immunohistochemistry using the antibodies anti-p75NTR (A, D, and G) and double staining with the antibodies antilaminin and antineurofilament (B, E, and H) of the normal group (A and B), TPCL (D and E), and TPCLF (G and H), 60 days postsurgery. Scale = 100 μm. The quantification of the integrated density of pixels for p75NTR reveal a greater immunoreactivity in the TPCLF group in comparison to the TPCL and normal (C). Laminin immunostaining quantification revealed upregulation in TPCL and TPCLF groups in comparison to normal. No quantitative differences were obtained for neurofilament immunostaining.

The anti-p75NTR antibody labeling was more intense for the TPCL and TPCLF groups as compared to the normal nerves (*P* < 0.001 and *P* < 0.001, respectively; Normal – 816.74 ± 137.68; TPCL – 6,675.88 ± 420.71; TPCLF – 9,789.59 ± 343.78, integrated density of pixels, mean ± SEM; Fig. 9). A comparison between the two experimental groups treated using the tubulization technique revealed greater labeling in the collagen implanted group (*P* < 0.001), consistent with the morphometrical data.

Immunolabeling against collagen IV did not reveal any noticeable differences amongst the experimental groups and the normal nerves. The same was observed for neurofilament staining (Figs. 7 and 8). Laminin immunoreactivity, on the other hand, was significantly weaker in the normal nerves and equally intense in the TPCL and TPCLF groups (Normal – 9,416.03 ± 863.05; TPCL – 14,141.60 ± 535.10; TPCLF – 14,495.82 ± 450.89, integrated density of pixels, mean ± SEM, *P* > 0.01).

### Polarizing microscopy

**Figure 10:**
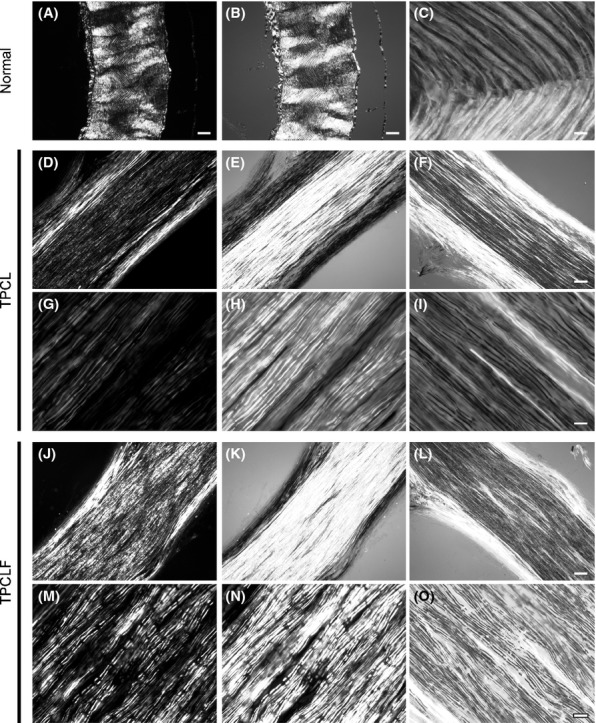
Analysis of the sciatic nerve birefringence under polarization microscope for the normal (A–C), TPCL (D–I), and TPCLF (J–O) groups. Birefringence of the nerve as a whole (A, D, G, J, and M); compensation of the birefringence of the collagen (B, C, E, H, K, and N); and compensation of the birefringence of the myelin (B, C, F, I, L, and O). Scale = 10 μm (overview images) and 100 μm (greater magnification).

For the longitudinal sections, polarizing microscopy revealed the presence of a highly birefringent pattern in the peripheral nerve. The normal nerve showed a wavy pattern of fibers demonstrating alternately bright and dark regions. This was consistent with the disposition of collagen fibers around myelinated axons, and indicated opposed patterns of diverted polarized light, so that when the birefringence of the collagen was compensated, the myelin sheath was revealed and vice versa. This suggests that, the normal peripheral nerves have a supra-organization, and that the distensibility of the conjunctive tissue surrounding the axons lead to the formation of collagen crimps. The wavy pattern of the nerve as a whole indicated that the collagen fibers were opposed in a perpendicular way to the compacted lipids present in the myelin sheath. This contributed to the formation of a helical three-dimensional structure. The present results indicated, for the first time, that the suprastructure of the nerve resulted from the presence of highly organized molecules within the endoneural sheath, and could be accessed by polarization microscopy (Fig. 10).

Interestingly, the TPCL group samples showed fibers that were arranged in a less parallel fashion as compared to the normal nerve. Thus, the birefringence of the collagen and myelin could be better depicted close to the epineurium. When the birefringence of the collagen was compensated, dark regions forming large bundles could be seen, and were interpreted as the presence of extracellular matrix molecules associated with regenerating fibers. By compensating the birefringence of the myelin, this correlation became more evident by the increasing brightness of the collagen.

For the TPCLF group, the organization of the nerve was similar to that of the group described above, although the amplitude of the collagen birefringence of these samples was greater and the fibers presented a more compact disposition, indicating a pattern closer to that of the normal nerve. The collagen fibers were distributed in smaller bundles within the nerve in a homogeneous way intermingled with other endoneural nerve elements. This could be made more evident by compensating the birefringence of the myelin, resulting in a slightly brighter image of the nerve as a whole. The more aggregated disposition of these elements, as a result of the implant with the collagen with a supra-molecular organization, reinforced the positive role on the Schwann cells during the regenerative process.

## Discussion

For years, the tubulization technique has been studied in an attempt to better understand the regenerative process, and in some cases, to replace the autograft approach (Fields et al. 1989; Yannas and Hill 2004; Pierucci et al. 2009). Tubulization allows for the use of molecules from the extracellular matrix on the inside of the tube, in order to optimize peripheral nerve regeneration. These strategies have shown promising results, positively influencing angiogenesis and leading to proliferation, migration, and differentiation of the Schwann cells (Keilhoff et al. 2003; Badylak et al. 2009).

The architecture and development of biological implants are in constant evolution, starting from an inert mechanical support and progressing to a dynamic platform for adhesion, proliferation, differentiation, and cell interaction with the physiological microenvironment (Verdú et al. 2002; Yow et al. 2009; Kijeńska et al. 2012; Wang et al. 2012).

There is a general consensus that nerve regeneration is improved when implants of extracellular matrix are aligned along the tube axis. The orientation facilitates elongation of growth cones, avoiding neuroma formation (Dubey et al. 1999).

In tubes filled with aligned implants, the regeneration of fibers can be guided in a contact-oriented fashion (Verdú et al. 2002). The physical and chemical properties of the microenvironment are crucial for axonal regeneration and the interaction between regenerating axons and the adjacent substrate can be a key factor in axonal elongation (Alluin et al. 2009).

Oliveira et al. (2005) considered that the organization and molecular aggregation state of the collagen in nerve implants were important factors that provided a suitable environment for regeneration and axon orientation. Naturally oriented proteins can facilitate axonal sprouting and be degraded more efficiently. In this sense the collagen with a supra-molecular organization extracted from the bovine tendon showed a supra-structural pattern consisting of oriented fibers.

In research carried out by Ceballos et al. (1999), a magnetically aligned collagen gel was studied on the inside of a nerve guide, to improve peripheral nerve regeneration. The samples of nerves regenerated with the magnetically aligned gel showed the formation of nerve fascicles and, despite the fact that the number of myelinated fibers was smaller than that of the normal nerve it was possible to note the rapid reabsorption of the collagen implant.

By contrast, it was considered that the collagen subjected to different treatments could not be presented in the self-organized form into the oriented helical fibers (Oliveira et al. 2005). However, as a result of specific methods of production, the collagen with supra-molecular organization used in this work did not present changes in its superstructure, and thus maintained the natural organization of the fibers/bundles in its helical arrangement.

Supporting the concept that an aligned implant favors peripheral nerve regeneration, Hadlock et al. (2000) used a collagen implant longitudinally oriented by 5-associated channels to introduce Schwann cells using the tubulization technique. The material was implanted in a 7-mm area (similar to the gap used in the present work) between the stumps of the transected sciatic nerve of Fisher rats. The axonal regeneration was assessed in the central region of the tube 6 weeks postoperatively and compared with the autograft. As a result, the percentage of nerve tissue present in the cross-sectional area of the group with collagen implants with channels was greater than that of the autograft.

Interestingly, the results herein are particularly different in this regard, so that the number of axons found in the AG group was nearly twice as much the TPCL and TPCLF groups. However, apart from the greater number of axons, myelin thickness and “g” ratio were better in the collagen implanted group. This indicates that the implant may direct the growing axons more efficiently, decreasing the need of fiber sprouting. This may in turn improve the myelination process, as Schwann cells will associate with less and larger axons. Such hypothesis is also in line with the quantification of p75NTR and laminin immunoreactivity.

Using the magnetically aligned collagen gel, Ceballos et al. (1999) analyzed a 60-day regeneration period in mice. The average values obtained for the cross-sectional areas of nerves regenerated with the implant of a magnetically aligned collagen gel, was higher than in the groups with a nonorganized implant. The regenerated myelinated fiber count was significantly higher in the presence of the aligned collagen, as compared to the control groups. However, the thickness of the myelin sheath was reduced in comparison to the groups without the aligned gel.

The present data are in line with the results described by Ceballos et al. (1999) with regard to the nerve area and the number of regenerated fibers. Nevertheless, an improved myelin thickness was found with the implant of collagen with a supra-molecular organization (TPCLF). This could also be noticed in the ultrastructural findings as well as by polarizing microscopy. The present results additionally revealed, together with the increase in thickness of the myelin sheath, that the supra-organized collagen implant favored a close to normal extracellular matrix reorganization during the regeneration process. It is important to emphasize that within the first hours following tubulization, the gap between the stumps is filled with a fluid that is rich in growth-supporting factors. The presence of the aligned collagen implant may have facilitated the retention of such substances, what may in turn stimulate cell migration into the structured scaffold. The acceleration of the initial steps of the regenerative process may lead to the improvement of myelination and morphological characteristics described herein. In addition, due to the structural support given by the collagen implant with a supra-molecular organization, the migration of Schwann cells could be anticipated and optimized. This is also supported by the immunohistochemical data regarding the expression of p75NTR.

The use of polarization microscopy revealed important new features of the normal nerve, such as the wavy supra-organization (crimping of collagen fibers), which is similar to that described for tendons and tendinous cords of the atrioventricular valves of the heart (Vidal 2003; Vidal and Mello 2008; Vidal and Mello 2009). This indicates that the collagen organization in the microenvironment of peripheral nerves provides, besides its structural role, a scaffold for the alignment of the axons within the nerve bundle. Thus, it is believed that the use of collagen with a supra-molecular organization facilitates the repair of the microenvironment of the nerve, resulting in more compact and organized mini-fascicles.

Taking into account the discussed above, the authors believe that the tubulization technique associated with the use of naturally organized molecules of the extracellular matrix is an acceptable approach for peripheral nerve repair. The regenerative process associated with the supra-molecular organized collagen provided a dynamic environment, allowing for axonal regeneration and the proper reorganization of the extracellular matrix in a more close to normal fashion. This is desirable in order to re-establish nerve homeostasis and function. Nevertheless, further investigations focusing on the functional recovery will be necessary in order to support the present findings.

Other studies are underway in the same laboratory to further improve the use of the tubulization technique associated with self-assembling molecules with supra-organization, associated with other stimulating elements such as neurotrophic factors and stem cells.

## Supporting Information

**Movie S1.** 3D electron tomography reconstruction of the collagen implant obtained in a transmission electron microscope (BioTwin G^2^ Spirit, FEI Company, The Netherlands). The confocal images series is followed by the volume rendering showing the parallel organization of the collagen fibrils.

## References

[b1] Alluin O, Wittmann C, Marqueste T, Chabas JF, Garcia S, Lavaut MN (2009). Functional recovery after peripheral nerve injury and implantation of a collagen guide. Biomaterials.

[b2] Badylak SF, Freytes DO, Gilbert TW (2009). Extracellular matrix as a biological scaffold material: structure and function. Acta Biomater.

[b3] Ceballos D, Navarro X, Dubey N, Wendelschafer-grabb G, Kennedy WR, Tranquillo RT (1999). Magnetically aligned collagen gel filling a collagen nerve guide improve peripheral nerve regeneration. Exp. Neurol.

[b4] Dubey N, Letourneau PC, Tranquillo RT (1999). Guided neurite elongation and Schwann cell invasion into magnetically aligned collagen in simulated peripheral nerve regeneration. Exp. Neurol.

[b5] Evans GR, Brandt K, Widmer MS, Lu L, Meszlenyi RK, Gupta PK (1999). In vivo evaluation of poly (L-lactic acid) porous conduits for peripheral nerve regeneration. Biomaterials.

[b6] Fields RD, Longo JM, Le Beau FM, Ellisman MH (1989). Nerve regeneration through artificial tubular implants. Prog. Neurobiol.

[b7] Hadlock T, Sundback C, Hunter D, Cheney M, Vacanti JP (2000). A polymer foam conduit seeded with Schwann cells promotes guided peripheral nerve regeneration. Tissue Eng.

[b8] Karlsson M, Johansson F, Kanje M (2011). Polystyrene replicas of neuronal basal lamina act as excellent guides for regenerating neurites. Acta Biomater.

[b9] Keilhoff G, Stang F, Wolf G, Fansa H (2003). Bio- compatibility of type I/III collagen matrix for peripheral nerve reconstruction. Biomaterials.

[b10] Kijeńska E, Prabhakaran MP, Swieszkowski W, Kurzydlowski KJ, Ramakrishna S (2012). Electrospun bio-composite P(LLA-CL)/collagen I/collagen III scaffolds for nerve tissue engineering. J. Biomed. Mater. Res. B Appl. Biomater.

[b11] Labrador RO, Butí M, Navarro X (1998). Influence of collagen and laminin gels concentration nerve regeneration after resection and tube repair. Exp. Neurol.

[b12] Lohmeyer JA, Siemers F, Machens HG, Mailänder P (2009). The clinical use of artificial nerve conduits for digital nerve repair: a prospective cohort study and literature review. J. Reconstr. Microsurg.

[b13] Lu Q, Simionescu A, Vyavahare N (2005). Novel capillary channel fiber scaffolds for guided tissue engineering. Acta Biomater.

[b14] Lundborg G, Rosen B, Abrahamson SO, Dahlin L, Danielsen N (1994). Tubular repair of the median nerve in the human forearm. Preliminary findings. J. Hand Surg.

[b15] Lundborg G, Rosen L, Dahlin L, Holmberg J, Rosen I (2004). Tubular repair of the median or ulnar nerve in the human forearm: a 5-year follow-up. J. Hand Surg.

[b16] Mayhew TM, Sharma AK (1984). Sampling schemes for estimating nerve fibre size. II. Methods for unifascicular nerve trunks. J. Anat.

[b17] Oliveira ALR, Pierucci A, Pereira KB (2004). Review: peripheral nerve through the nerve tubulization technique. Braz. J. Morphol. Sci.

[b18] Oliveira ALR, Vidal BC, Langone F (2005). Naturally supraorganized collagen increases axonal regeneration after tubulization repair. Braz. J. Morphol. Sci.

[b19] Pierucci A, Duek EAR, Oliveira ALR (2008). Peripheral nerve regeneration through biodegradable conduits prepared using solvent evaporation. Tissue Eng.

[b20] Pierucci A, Duek EAR, Oliveira ALR (2009). Expression of basal lamina components by Schwann cells cultured on poly (lactic acid) (PLLA) and poly (caprolactone) (PCL) membranes. J. Mater. Sci. Mater. Med.

[b21] Ribeiro-Resende VT, Koenig B, Nichterwitz S, Oberhoffner S, Schlosshauer B (2009). Strategies for inducing the formation of bands of Büngner in peripheral nerve regeneration. Biomaterials.

[b22] Verdú E, Labrador RO, Rodríguez FJ, Ceballos D, Forés J, Navarro X (2002). Alignment of collagen and laminin-containing gels improve nerve regeneration within silicone tubes. Restor. Neurol. Neurosci.

[b23] Vidal BC (1995). From collagen type I solution to fibers with helical pattern: a self assemble phenomenon. C. R. Acad. Sci. III.

[b24] Vidal BC (2003). Image analysis for tendon helical superstructure using interference and polarized light microscopy. Micron.

[b25] Vidal BC (2010). Form birefringence as applied to biopolymer and inorganic material supraorganization. Biotech. Histochem.

[b26] Vidal BC, Mello MLS (2009). Structural of collagen fibers in chordate tendineae as assessed by optical anisotropic properties and fast fourier transform. J. Struct. Biol.

[b27] Vidal BC, Mello MLS (2010). Optical anisotropy of collagen fibers of rat calcaneal tendons: an approach to spatially resolved supramolecular organization. Acta Histochem.

[b28] Vidal BC, Mello MLS, Caseiro-Filho AC, Godo C (1980). Anisotropic properties of the myelin sheath. Acta Histochem.

[b29] Wang CY, Liu JJ, Fan CY, Mo XM, Ruan HJ, Li FF (2012). The effect of aligned core-shell nanofibres delivering NGF on the promotion of sciatic nerve regeneration. J. Biomater. Sci. Polym. Ed.

[b30] Yannas IV, Hill BJ (2004). Selection of biomaterials for peripheral nerve regeneration using data from the nerve chamber model. Biomaterials.

[b31] Yow SZ, Quek CH, Yim EK, Lim CT, Leong KW (2009). Collagen-based fibrous scaffold for spatial organization of encapsulated and seeded human mesenchymal stem cells. Biomaterials.

