# Supplementary material for: Enhanced peripheral nerve regeneration by the combination of a polycaprolactone tubular prosthesis and a scaffold of collagen with supramolecular organization
Source: Brain Behav. 2013 May 30;3(4):417–30. doi: 10.1002/brb3.145 (PMC3869682; doi:10.1002/brb3.145)
Supplement: Supplementary file 1 [file brb30003-0417-SD1.docx]

**Movie.avi** – 3D electron tomography reconstruction of the collagen implant obtained in a transmission electron microscope (BioTwin G^2^ Spirit, FEI Company, The Netherlands). The confocal images series is followed by the volume rendering showing the parallel organization of the collagen fibrils.
